# Measuring Suite for Vascular Response Monitoring during Hyperbaric Oxygen Therapy by Means of Pulse Transit Time (PTT) Analysis

**DOI:** 10.3390/s22218295

**Published:** 2022-10-29

**Authors:** Theresa Wandel, Daniel Pascal Hausherr, Dirk Berben

**Affiliations:** Physics Laboratory, Campus Hagen, South Westphalia University of Applied Sciences, Haldener Str. 182, D-58095 Hagen, Germany

**Keywords:** wearable biomedical sensing, human health monitoring, pulse transit time evaluation

## Abstract

The efficacy of hyperbaric oxygen therapy in treating wound healing disorders is well established. The obvious explanation is the presence of elevated oxygen tissue tensions during the high-pressure oxygen exposure. This explanation omits that the effective agent, elevated oxygen tension, is only present for 6.25% of the time. To investigate possible prevailing vascular changes caused by HBOT, the presented device monitors the vascular response during therapy by Pulse-Transit-Time analysis. The device allows synchronous 1 kHz ECG and PPG measurements. The data are stored in a 1 GBit flash drive and retrieved post-therapy. Normoxic measurements on the authors with and without nicotine validate the device’s functionality. Measurements during HBO therapy have been successfully performed.

## 1. Introduction

In humans, oxygen plays a crucial role in providing energy and sustaining life and functionality [1]. A variety of diseases and defects correlate with a lack of oxygen supply (hypoxia) that can lead to severe impairment and even death [2]. In the late eighteenth century, a therapy was developed, which counteracts the effects of hypoxia and decompression sickness: hyperbaric oxygen therapy (HBOT) [2]. The treatment involves the intake of pure oxygen under increased ambient pressure, hence the term hyperbaric, thereby increasing oxygen intake (hyperoxia) and enhancing oxygen tissue tension throughout the body. The therapy is now widely used in medical treatment of various maladies such as wound healing disorders, carbon monoxide poisoning, or decompression sickness [3,4] and the understanding of its working mechanisms continues to improve [2]. The duration and utilized pressure in HBOT depends on the diagnosis and intended therapeutic result. For the most prevalent application in the treatment of wound healing disorders, the standard parameters are HBOT given at 2.0 to 2.4 ATA for 90 minutes daily for 30 to 40 days [5,6,7,8].

**Figure 1:**
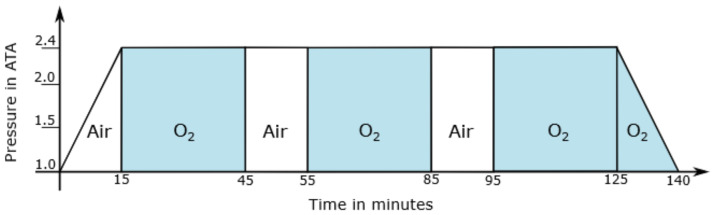
Diagram of the HBO therapy scheme TS 240-90 [3,4]. Air is defined as normal ambient air. Oxygen (O_2_) represents the administration of pure oxygen. The ordinate shows the pressure in atmospheres absolute, the abscissa shows the treatment time in minutes.

In order to avoid the Paul Bert effect in patients, gas- or air-break episodes are introduced. Figure 1 illustrates the therapy scheme TS 240-90 [3] that is divided into different sections:

1. Compression to 2.4 Atmospheres Absolute (ATA);
2. Isopressure phase during which pure oxygen is administered for 30 min via respiratory masks with a subsequent 10 min ambient air break, each repeated twice;
3. Isopressure phase during which pure oxygen is administered for 30 min via respiratory masks with subsequent decompression back to normal ambient air (1 ATA) with continued oxygen exposure

It is commonly assumed that hyperbaric oxygen (HBO) has a vasoconstrictive effect on human arteries [1,2,9,10,11,12]. Vasoconstrictive effects can be detected by studying changes in the Pulse Transit Time (PTT). The propagation of the pulse pressure wave in an artery depends, inter alia, on the diameter *D* and the elasticity *E* of the vessel, both of which are affected by vasoconstriction. The Pulse Wave Velocity (PWV ) [13,14,15,16] as described by the Moens–Korteweg equation

(1) $$PWV=\sqrt{\frac{Eh}{\rho D}},$$

increases as a result to HBO exposure, as the diameter *D* decreases and elasticity *E* increases, and can thus be used as an indicator for the cardiovascular response to HBO. Here, PWV refers to the Pulse Wave Velocity, *E* to Young’s modulus of elasticity, *h* to a vessel’s wall thickness, *D* to the diameter of the artery, and ρ to the blood density.

The PWV affects the time period required for the pulse-generated pressure wave to travel between two arterial sites, e.g., between heart and finger, within the same cardiac cycle. This is referred to as the PTT and it is directly proportional to the state of the cardiovascular system. Consequently, arterial stiffness, vasoconstriction and systemic factors, such as age or cardiovascular health, which affect the elasticity of the arteries, produce changes in PTT. We can therefore predict a shortened PTT as a result of HBO exposure [9,10,11,12,17,18]. Most studies on the effect of hyperbaric oxygen on the vascular system focus either on young healthy volunteers or have very limited participant counts. While devices to measure the PTT are commercially available, most hyperbaric chambers are equipped with one such device, if at all, due to cost limits. The measuring unit presented in this article, named SPECTRE 3.0, is designed as an affordable tool to study the above described effects of HBO on the vascular system on a larger patient number. SPECTRE 3.0 is currently in use in a clinical trial at the HBO-center of the University Clinic of Duesseldorf. The results of this ongoing study are to be presented at a later time and are thus not considered in this article.

## 2. Materials and Methods

**Figure 2:**
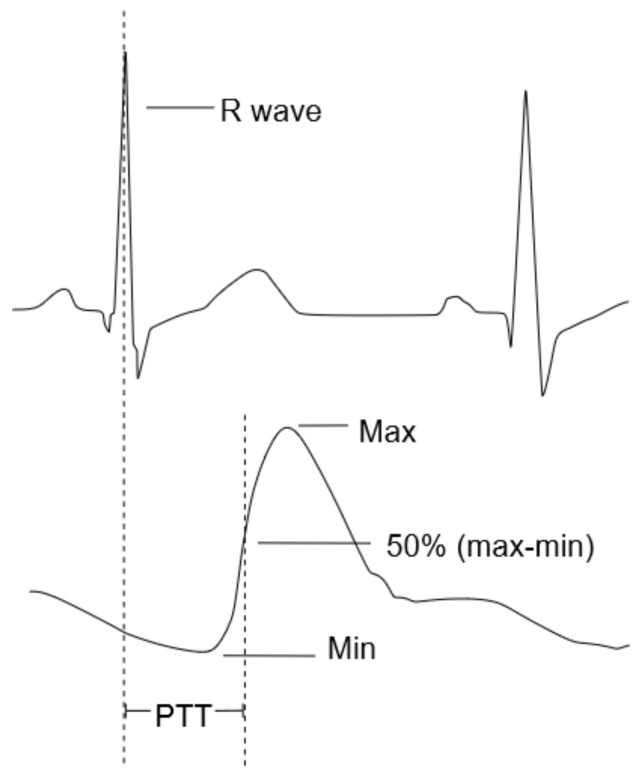
Graphical illustration of the Pulse Transit Time (PTT) using an electrocardiographic (**above**) and photoplethysmographic (**below**) signal.

The PTT is typically measured by detecting the time of the R-peak of an Electrocardiogram (ECG) as starting point, which is closely related to the opening of the aortic valve [17], and measuring the arrival of the pulse wave at a peripheral site, e.g., the finger, using a Photoplethysmogram (PPG) [19]. Figure 2 graphically illustrates the PTT as the time difference between the R-peak of the ECG signal and the inflection point of the PPG signal.

### 2.1. Sensor Selection

The correct evaluation of the PTT requires synchronized recordings of ECG and PPG and a data acquisition rate of approximately 1 kHz. SPECTRE 3.0 is equipped with a Texas Instruments four-channel AFE4900 analog-front-end (AFE) that has the ability to synchronously acquire several different biosignals, i.e., one ECG and up to three PPG signals. The sensor therefore allows the recording of PPG and Peripheral Oxygen Saturation (SpO_2_ ) data. PPG data acquisition is achieved by measuring changes in green LED light absorbance via Photodetectors (PDs). To allow parallel measurement of SpO_2_, another two LEDs are needed—one red and one infrared (IR) LED. For this purpose, with selected OSRAM’s SFH7072, a multichip package that provides two green (526 nm) emitters, one red (660 nm) emitter, one IR (950 nm) emitter, one broadband PD, and one IR PD. It is also optimized for PPG signal acquisition in wearable devices. The AFE4900 utilizes different time slots for each of the three LEDs during which one LED is turned on and the resulting PD signal is recorded. One full sample thus contains four datasets consisting of one PPG-signal for each LED and one ECG-signal.

The pressure chamber can induce claustrophobia and is generally, at least during the first few exposures, considered stressful for patients. While high-speed datatransfer of PTT data out of the chamber was rejected, the metal pressure chamber acts as a Faraday cage, low-speed data transfer is possible. Therefore, an additional sensor is added to continuously monitor the patient’s heart rate during HBOT and transmit this information to the chamber operator via the control station outside the chamber. This allows medical staff to notice early signs of distress and to intervene in a timely manner. The *MAX30003* by Maxim Integrated is an integrated biopotential AFE for wearable applications that is capable of R-to-R detection in an ECG-signal and thus quickly computes the momentary heart rate.

### 2.2. Hardware Design

**Figure 3:**
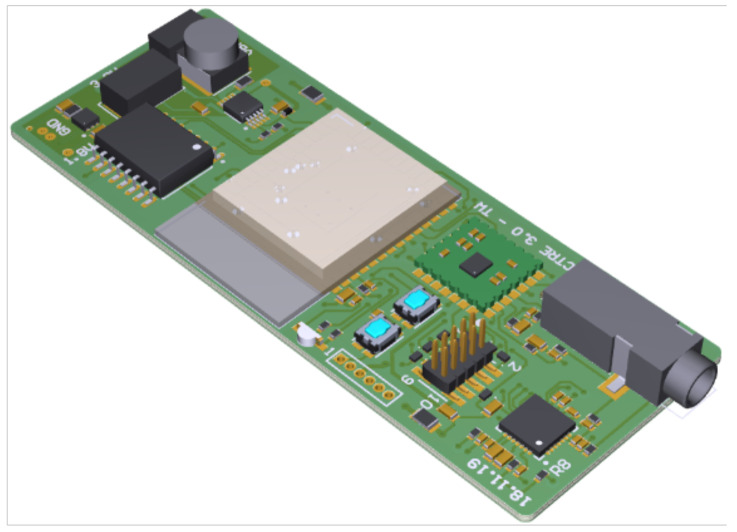
3D model of the 28 ×73 mm${}^{2}$ measuring unit PCB.

A 28 × 73 mm${}^{2}$ printed circuit board (PCB) is designed that hosts the bulk of the circuitry (shown in Figure 3), namely, a microcontroller unit (MCU) for controlling the system, a programming interface, flash-memory for recorded data, power-supply via batteries, electrode and sensor connectors, the AFE4900, and the MAX30003. The system’s integrated flash memory, Cypress Semiconductor Corporation’s S70FL01GS, provides a sufficient amount of memory (1 Gbit in total) to store the entirety of the data generated by the AFE4900 during a two-hour therapy session. Another smaller PCB with a diameter of 15 mm hosts the PPG sensor LEDs and PDs, can be mounted on the finger, and is connected to the main board with a suitable ribbon cable and pin header connector.

The AFE4900 and MAX30003 receive the ECG signal from a three-lead ECG electrode audio jack connector. The ECG signal is collected from the human body, with the help of adhesive electrodes in an Einthoven configuration on the left and right forearm of the patient, and transferred to the connector on the main PCB via two cable electrodes. The AFE4900 has an additional Right Leg Drive (RLD) amplifier output, which is connected to a third ECG cable electrode and used to bias the patient to a fixed 0.9 V direct current (DC) operating point to ensure a defined common mode of the ECG signal suitable for the AFE’s operating conditions. Before entering the AFE4900, the ECG-signal is filtered through a passive analog first-order *RC* low-pass filter (LPF) to reduce high-frequency noise on the ECG-signal such as potentially coupled 50 Hz power line noise.

SPECTRE 3.0’s power is supplied by two AAA alkaline batteries connected in series on a battery holder on the bottom side of the main PCB. This integrated battery holder facilitates quick battery (re-)placement without cumbersome wire connections. A piece of circuitry is added that allows SPECTRE 3.0 to be switched off by using a detachable magnet. Once a magnet is placed near the PCB’s reed sensor, SPECTRE 3.0 is powered off by disconnecting the device from the battery power. Withdrawing the magnet restores power supply and SPECTRE 3.0 is powered on. This approach allows the use of SPECTRE 3.0 even if no magnet is available. In this case, SPECTRE 3.0 can always be powered off by removing the batteries from the battery holder. The two batteries with 1300 mAh last for approximately 7 h of recording time and pose no danger of fire or other unwanted reactions to elevated ambient pressures.

**Figure 4:**
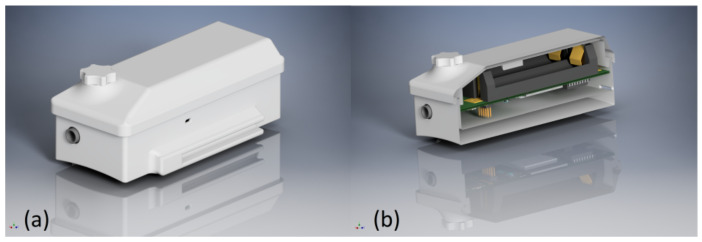
Housing of SPECTRE 3.0. (**a**) Exterior view of the device’s housing showing the assembled body and detachable lid and magnet holder. (**b**) Cross-section of the housing showing the placement of the PCB inside.

**Figure 5:**
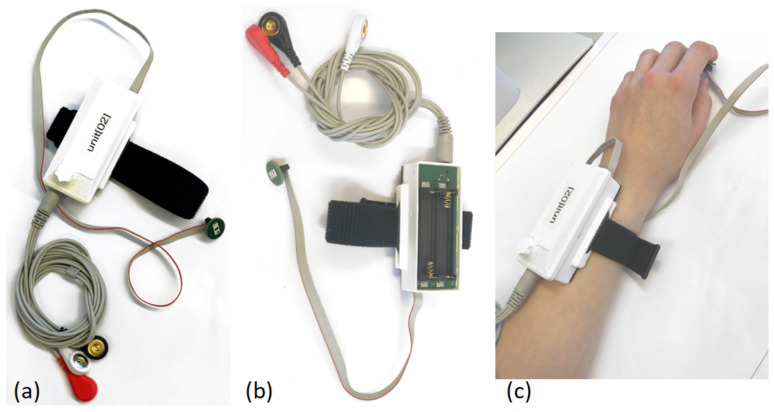
Wearable measuring unit with ECG electrodes, PPG finger sensor and a wrist strap. (**a**) Closed measuring unit with connected ECG electrodes and PPG sensor. (**b**) Measuring unit without the lid showing the battery connector’s accessibility. (**c**) SPECTRE 3.0 assembled on a person’s wrist, showing teh placement of the PPG finger sensor.

SPECTRE 3.0 is placed in a 3D-printed case that has a detachable lid to facilitate battery exchange, and it is shown in Figure 4. The housing incorporates recesses for connectors, cable routing, and indicator LEDs, as well as a fixture for attaching a wristband for easy application and a mount for the detachable magnet on top. Figure 5 shows pictures of the assembled device including the PPG sensor and ECG electrodes.

**Figure 6:**
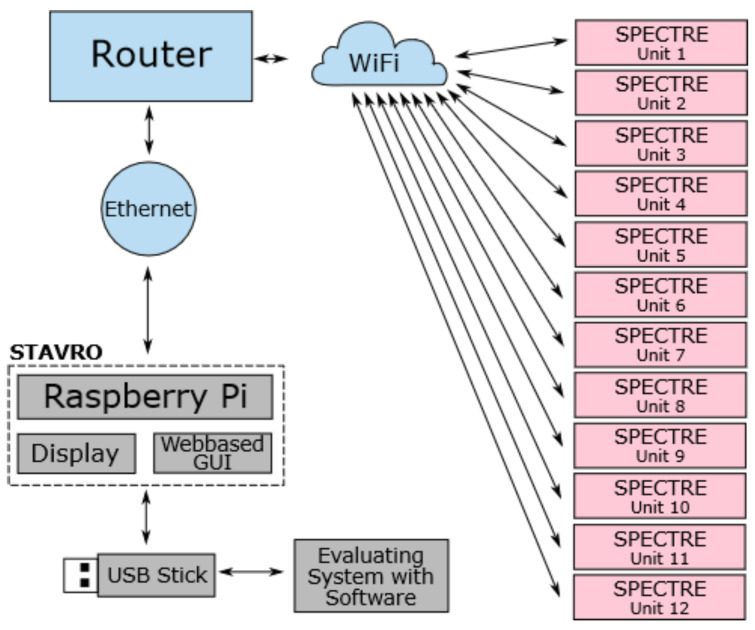
Overall system topology consisting of twelve SPECTRE 3.0 measuring units, one control unit, a router, and a data evaluating system running on a PC.

**Figure 7:**
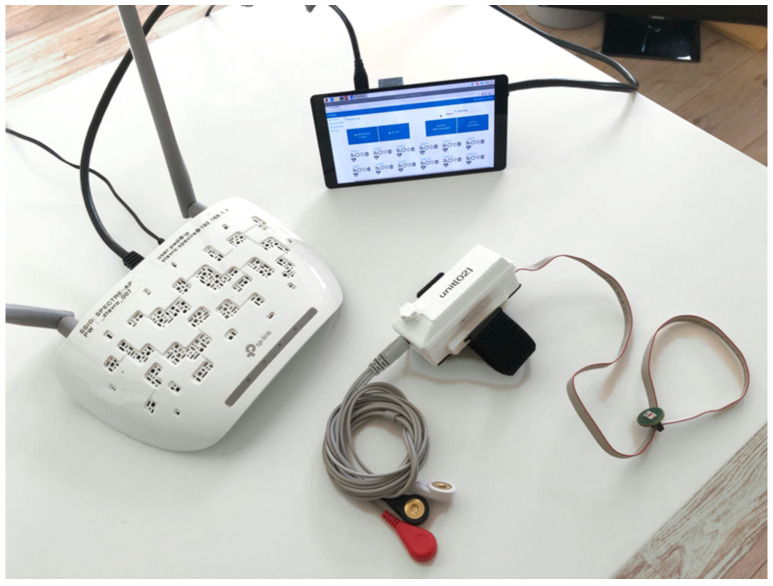
Systemsetup showing the control unit with touch display and graphical user interface, a router, and one SPECTRE 3.0 measuring unit.

The overall topology of the system is designed to allow data acquisition during HBOT and the subsequent evaluation of that data. The system consists of one control unit and up to twelve SPECTRE 3.0 measuring devices that are connected to a Wireless Local Area Network (WLAN) created by an access point, e.g., a router. Figure 6 illustrates this overall system topology with arrows demonstrating the directions of data transfer between the components. The control unit is a single board computer (Raspberry Pi 3 Model B+) with a touch display and a Graphical User Interface (GUI). Figure 7 shows a photograph of the complete setup consisting of the control unit, a router and a measuring device. The measuring units can be worn by patients inside the hyperbaric chamber during HBOT. The control unit remains outside of the chamber and is to be operated by the chamber personnel. Subsequent to HBOT monitoring, the raw data are first transferred from the individual measuring units to the control unit via WiFi. Afterwards, the data are transferred to an evaluating system, e.g., a personal computer (PC) running an evaluation software, via wireless connection or external storage devices.

### 2.3. Software Design

The measuring unit’s central MCU controls the system, initially processes and stores the data provided by the biosensors and communicates with the control unit. The communication relies on the MQTT protocol for transmitting requests and status information. SPECTRE 3.0 can receive *record data*, *send data*, and *delete data* requests from the control unit and it can send status and heart rate information to be displayed in the GUI. The heart rate information is calculated by using the MAX30003’s data on R-to-R intervals and transmitted to the control unit once per second. The requests *send data* and *delete data* are only available when SPECTRE 3.0 is not recording data. The delete flash command erases the entire flash memory storage. The send data command reads out the flash memory storage until the last entry and transmits the resulting data packages to the host via the Transmission Control Protocol (TCP). The data are sent in binary format and saved as a binary file for subsequent processing using the evaluation software. Furthermore, the chamber personnel can transmit the specific onset of oxygen administration to the measuring unit via the control unit by pressing a button in the device’s GUI. This allows SPECTRE 3.0 to add the actual time of oxygen onset to the data set to improve the accuracy of the results. In order to provide minimal and unobtrusive visual feedback for fast and reliable user-updates on the basic operational state of SPECTRE 3.0, the main PCB is equipped with a multi-colour LED indicator, whose state can be observed through an opening on the side of the housing. Information encoding is described in Table 1.

Most errors can be corrected by restarting the device, checking the WLAN network, or changing the batteries. Potential errors include:

- Battery power is too low for WLAN connection;
- Cannot connect to WLAN network;
- Cannot connect to MQTT broker (automatically attempts reconnect);
- Error while initialising any hardware component;
- Error while erasing the flash memory.

### 2.4. Data Processing and Evaluation

The recorded data are processed and evaluated using an appropriate evaluation software running on Windows Systems. The software is based on MATLAB^®^ algorithms for digitally processing and filtering ECG and PPG data. A subsequent analysis detects the ECG signal’s R-peaks and computes the time difference to the next PPG signal’s inflection point, thus computing the PTT’s progression during the measuring interval. Furthermore, the software evaluates the R-to-R intervals of the ECG signals, thereby computing heart rate progression as well as heart rate variability.

### 2.5. Detecting Changes in Pulse Transit Time during Nicotine Administration

To test the measuring method, the effects of nicotine intake on the cardiovascular system were measured using SPECTRE 3.0. Nicotine is known to have a vasoconstrictive effect and to increase the PWV [20,21,22,23]. We therefore expect a visible decrease in PTT during nicotine intake. The trial was performed on one of the authors as follows:

1. Volunteer rests for 5 min;
2. Volunteer smokes 3 cigarettes in quick succession (approx. 5 min);
3. Volunteer rests for another 10 min.

The data acquired with SPECTRE 3.0 during the trial were afterwards processed with the evaluating software to compute the PTT’s and heart rate’s progression over time.

## 3. Results

### 3.1. Validation of Measuring Methods

**Figure 8:**
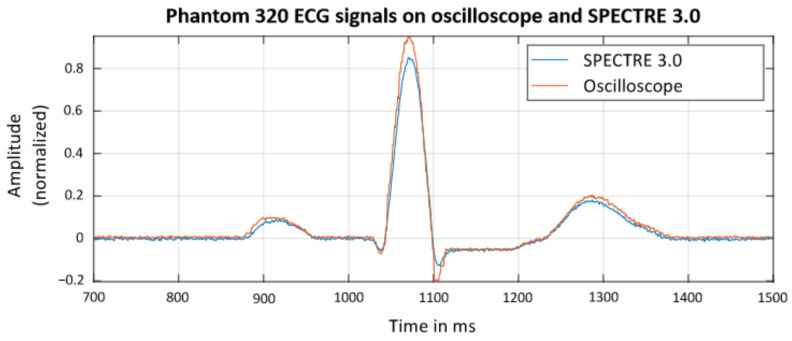
Comparisonbetween standardized PQRST-complexes simulated by the phantom 320 ECG simulator and recorded simultaneously with both SPECTRE 3.0’s AFE4900 and a 12 bit-oscilloscope.

The basic functionality of the device was validated by using a phantom 320 ECG simulator by the company Müller und Sebastiani Elektronik GmbH. The ECG was measured both with an oscilloscope and SPECTRE 3.0. The comparison is shown in Figure 8. The excellent agreement between SPECTRE 3.0’s data and the oscilloscope’s reference data confirms that SPECTRE 3.0 is able to obtain valid ECG data from human subjects.

**Figure 9:**
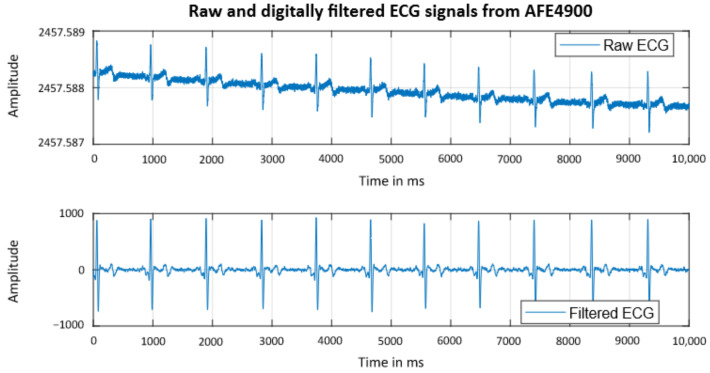
Raw (unfiltered) and digitally filtered ECG signal recorded with SPECTRE 3.0.

Additional tests are performed to test the ECG signal quality. The upper graph in Figure 9 shows the raw (unfiltered) ECG signal obtained with SPECTRE 3.0. from one of the authors. The signal shows a drift resulting from minor motion artifacts. Additional digital filtering using the evaluation software further enhances the signal by reducing noise, normalizing the signal, and eliminating trends and motion artifacts, as seen in Figure 9 in the graph below. The result is a signal with clearly visible R-peaks that allows subsequent PTT calculation.

The heart rate calculated with the MAX30003 data is validated by comparing the displayed value to other methods of heart rate evaluation. First, the ECG-simulator phantom 320 ECG simulator by Müller und Sebastiani Elektronik GmbH is connected to SPECTRE 3.0. The phantom 320 can output various ECG signals with different frequencies. SPECTRE 3.0’s displayed heart rate values match the configured phantom 320 ECG output frequencies. The value is consistent at lower frequencies (i.e., 60 beats per minute (BPM) and lower), whereas a small deviation (up to ±5%) can be observed in higher frequencies (i.e., 75 BPM +). Further tests on the authors are performed and the obtained heart rates are compared to the computed values of two other devices: the Apple Watch Series 5 and the Sanitas SPO25 finger pulse oximeter. These two devices were chosen as they were available in the laboratory and allowed for easy comparison of simultaneously recorded heart rate measurements on human subjects. In addition, we expect the Apple Watch Series 5 heart rate data to be reliable since its predecessor is the FDA-approved Apple Watch Series 4, which has been shown to be able to accurately detect heart rate and rhythm [24]. SPECTRE 3.0’s calculated heart rate values match the displayed values of the other two devices with deviations of up to ±10%. Motion artifacts are observed. The findings indicate that the system can reliably monitor the heart rate of patients while recording if the movement of the patient is limited.

**Figure 10:**
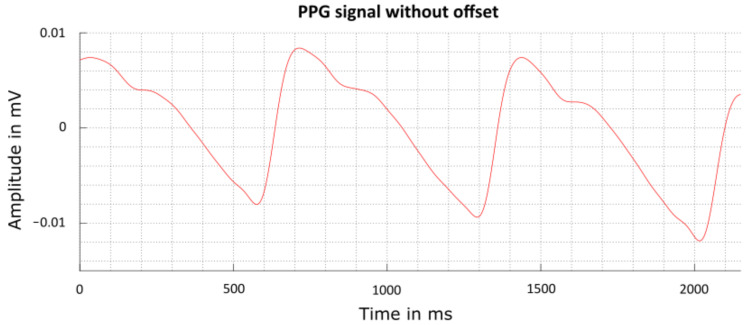
Digitally filtered PPG wave signal that was recorded with SPECTRE 3.0 and processed by the evaluation software.

The PPG signal recorded with SPECTRE 3.0 is compared to the characteristics of a pulse wave described in the work of Lax et al. [25] according to which a characteristic pulse wave is comprised of an initial wave and a lower dicrotic wave. The initial wave reaches its peak “within 0.1 to 0.15 s of the start of the wave” [25]. The dicrotic wave generally appears “from 0.35 to 0.45 s after the start of the initial wave” [25]. Similar intervals for the specified time periods can be observed in the PPG signals recorded with SPECTRE 3.0 shown in Figure 10. SPECTRE 3.0’s PPG signals are therefore considered reliable for determining the PPG’s inflection point and consequently calculating the PTT.

### 3.2. Nicotine Administration

**Figure 11:**
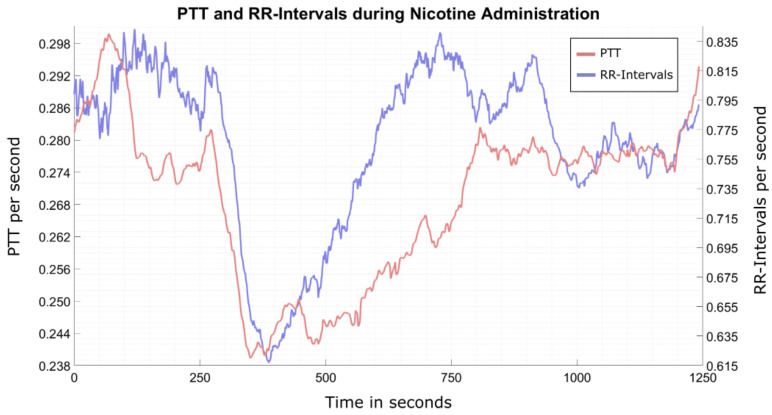
Effects of nicotine on the PTT. The data set recorded with SPECTRE 3.0 shows the PTT and R-to-R interval (heart rate) progression over time. Nicotine intake starts at 300 s and ends at approximately 600 s.

The PTT progression over time during nicotine administration shown in Figure 11 displays a distinct drop at the onset of nicotine intake at 300 s and recovers shortly after discontinuing substance administration at approx. 600 s. As a by-product of this measurement, we determined the pharmacokinetic profile of inhaled nicotine with regard to the PTT to be in the range of $\tau_{PTT}\approx$ 250 ms and $\tau_{RR}\approx$ 400 ms. The relaxation time constant of the heart rate (R-to-R intervals) is in excellent agreement with the decline of the mean plasma nicotine concentration after cigarette consumption [26], whereas the relaxation of the PTT is slightly faster. The vasoconstrictive effects of nicotine administration are clearly visible in the recorded data set. We therefore conclude that SPECTRE 3.0 is suitable for detecting changes in the course of the PTT over time.

### 3.3. Recording during HBOT

The system was tested under hyperbaric conditions during a standard HBOT at the University Hospital Duesseldorf. Two of the authors were equipped with SPECTRE 3.0 during the therapy session and recordings were managed by the control unit outside the hyperbaric chamber. Excellent data could be obtained from the devices, both during the therapy session (live heart rate monitoring) and after subsequently processing the data using the evaluation software. SPECTRE 3.0 has therefore proven to be operational under hyperbaric conditions and during HBOT. Multiple SPECTRE 3.0 units are currently involved in a clinical trial at University Clinic Duesseldorf, Center for Hyperbaric Medicine, Clinic for Orthopedics and Trauma Surgery. Several units have undergone dozens of therapy session, sometimes to pressures of up to 6 ATA with minimal failure rates. Replacement of defect units is simple and affordable due to the low cost of each individual unit. Study results are to be published at a later time.

## 4. Discussion

The aim of this project was to develop an advanced measuring unit that facilitates the acquisition of data on the changes in pulse transit time during HBOT and thereby enable researches to further examine the effects of hyperbaric oxygen on the vascular system. We should note, however, that the accuracy of the PTT evaluation results is highly affected by the quality of the captured ECG and PPG signals. Testing phases in the device’s developmental state showed that signal strength varies strongly between individuals. Subsequent processing and filtering of the recorded data is thus essential for obtaining reliable results.

The duration during which recordings can be obtained is limited by the devices battery capacity and the memory size. Although two AAA batteries with 1300 mAh are sufficient for ensuring a measuring period of up to 7 h, the memory size shortens this period significantly. With a storage capacity of 1 Gbit, the memory lasts only for approximately 2 h of recording time and is therefore sufficient for measurements during the aforementioned TS 240-90 therapy scheme. Currently, SPECTRE 3.0’s storage size might not provide full coverage for other therapy schemes requiring longer periods of HBO exposure. Future device enhancements could therefore include optimized data processing and compression directly on the microcontroller to better take advantage of the memory storage and expand the available recording period. Additionally, the data are not deleted automatically once a new recording is started. On the one hand, this prevents accidental deletion of data, but, in turn, it requires the device operator to actively clear the memory space in advance of a new measurement. In general, the device requires some attention from the chamber personnel when recording data as the onset of oxygen administration has to be marked actively using the control unit. In turn, the device offers additional monitoring of the patients by displaying the individuals’ momentary heart rate. However, this feature is still in need of improvement as a patient’s movement greatly affects the accuracy of the measurements.

Furthermore, even though the device allows recording of red and IR LED light absorption, the evaluation of these data with regard to monitoring SpO_2_ is currently not implemented due to lack of relevance. The SpO_2_ of all but the most severe cases of lung dysfunction in humans during HBOT is stable around 100%. The main objective of measuring changes in pulse transit time could be reached and demonstrated during trials using nicotine to create vascular responses.

## 5. Conclusions

The 28 × 73 mm${}^{2}$ small measuring unit, SPECTRE 3.0 is operational under hyperbaric conditions and records ECG and PPG data synchronously with a sample frequency of 1 kHz. These data can be subsequently used to calculate the current PTT and heart rate with great precision. Additional data can be acquired that allow the evaluation of blood oxygen saturation (SpO_2_). Moreover, SPECTRE 3.0 reliably computes the subject’s heart rate and, every second, transmits these data to the control unit outside the hyperbaric chamber. There, the heart rate is displayed in a GUI and thus allows live monitoring of the patients by the chamber personnel.

## Figures and Tables

**Table 1:** SPECTRE 3.0 indicator LED behaviour in relation to the device state.

Device State	LED_red_	LED_green_
No Connection to Network	ON	OFF
Connected to Network	OFF	ON
Deleting Flash	Blinking (T = 3 s)	-
Recording Data	Blinking (T = 1 s)	-
Sending Data	Blinking (T ≈ 0.4 s)	ON
Error	ON	ON

## Data Availability

The supporting firmware of the measuring unit and control unit is available at https://github.com/BerbenLabs/SPECTRE. The evaluation software is available from the corresponding author upon reasonable request.

## Abbreviations

The following abbreviations are used in this manuscript:

HBO	Hyperbaric Oxygen
HBOT	Hyperbaric Oxygen Therapy
PTT	Pulse Transit Time
PWV	Pulse Wave Velocity
ECG	Electrogardiogram
PPG	Photoplethysmogram
LPF	Low-Pass Filter
ATA	Atmosphere Absolute
SpO_2_	Peripheral Oxygen Saturation
LED	Light Emitting Diode
PD	Photodetector
IR	Infrared
AFE	Analog Front-End
PCB	Printed Circuit Board
MCU	Microcontroller Unit
DC	Direct Current
WLAN	Wireless Local Area Network
GUI	Graphical User Interface
PC	Personal Computer
BPM	Beats per minute
MQTT	Message Queuing Telemetry Transport
TCP	Transmission Control Protocol
